# The 4-Aminopyridine Model of Acute Seizures *in vitro* Elucidates Efficacy of New Antiepileptic Drugs

**DOI:** 10.3389/fnins.2019.00677

**Published:** 2019-06-27

**Authors:** Hanno Heuzeroth, Matthias Wawra, Pawel Fidzinski, Ramazan Dag, Martin Holtkamp

**Affiliations:** Epilepsy-Center Berlin-Brandenburg, Department of Neurology, Charité – Universitätsmedizin Berlin, Berlin, Germany

**Keywords:** 4-aminopyridine, levetiracetam, lacosamide, zonisamide, *in vitro*

## Abstract

Up to date, preclinical screening for new antiepileptic substances is performed by a combination of different *in vivo* models of acute seizures, for which large numbers of animals are necessary. So far, little attention has been paid to *in vitro* models, which are also able to detect antiepileptic efficacy and in principle could likewise serve for exploratory preclinical screening. One of the established *in vitro* models of acute seizures is the 4-aminopyridine (4-AP) model. Previous studies have shown that the 4-AP model is capable to recapitulate the antiepileptic efficacy of standard antiepileptic drugs (AEDs) such as valproate or carbamazepine. Here, we employed a dual methodological approach using electrophysiology and optical imaging to systematically test the antiepileptic efficacy of three new-generation AEDs with distinct mechanisms of action (lacosamide, zonisamide, and levetiracetam). We found that frequency of 4-AP induced seizure like events (SLE) was the most sensitive parameter to detect dose-dependent antiepileptic effects in these compounds. Specifically, levetiracetam reduced SLE frequency while lacosamide and zonisamide at higher doses completely blocked SLE incidence. Analysis of the intrinsic optical signal additionally revealed a subiculum-specific reduction of the area involved in the propagation of ictal activity when lacosamide or zonisamide were administered. Taken together, our data adds some evidence that acute seizure models *in vitro* are in principle capable to detect antiepileptic effects across different mechanisms of action with efficacy similar to acute models *in vivo*. Further studies with negative controls, e.g., penicillin as a proconvulsant, and other clinically relevant AEDs are needed to determine if this acute *in vitro* model might be useful as exploratory screening tool. In view of the increasing sensitivity toward animal welfare, an affective *in vitro* model may help to reduce the number of laboratory animals deployed in burdening *in vivo* experiments and to preselect substances for subsequent testing in time- and cost-laborious models of chronic epilepsy.

## Introduction

Epilepsy is a major neurological disorder affecting ∼1% of the population (Hirtz et al., 2007). Treatment of epilepsy is focused on reducing seizure burden, mainly achieved by antiepileptic drugs (AEDs). Driven by a significant pharmacoresistance rate of about 30% (Chen et al., 2018) and major adverse effects of classical AEDs like phenobarbital or carbamazepine (Perucca and Gilliam, 2012), several drug screening programs for detection of new AEDs were established in the last three to four decades (Löscher and Schmidt, 2011).

In these programs, initial screening of compounds with potential antiepileptic properties was mainly based on two acute *in vivo* seizure models: the maximal electroshock (MES) and the pentylenetetrazole model (PTZ). In the MES model introduced by Putnam and Merritt (1937), seizures are induced electrically by extracranial or corneal stimulation; MES-induced seizures are mainly sensitive to sodium channel blockers like carbamazepine or lamotrigine (Barton et al., 2001). The PTZ model, on the contrary, induces seizures chemically by blockade of GABA-A receptors (Löscher, 2011). Consequently, PTZ-induced seizures are rather sensitive to drugs acting on GABA-A receptors such as valproate or benzodiazepines (Everett and Richards, 1944; Barton et al., 2001). Although both models act in a complementary fashion, their combined ability to detect novel AEDs still has its weaknesses. This is best exemplified by levetiracetam, an AED introduced at the beginning of 21st century and widely used in clinical practice to date. Levetiracetam failed to be effective against seizures in both the MES and the PTZ models, but was effective against psychomotor seizures induced by 6 Hz corneal stimulation, another electrical model of acute seizures (Brown et al., 1953; Barton et al., 2001). Another important limitation is that *in vivo* models of acute seizures are implemented in naive brains which does not reflect the clinical setting in patients with chronic epilepsy characterized by and defined as an “enduring predisposition to generate epileptic seizures” (Fisher et al., 2014). Therefore in a second step, chronic epilepsy models, e.g., based on chemically induced status epilepticus by kainic acid or pilocarpine, and subsequent development of spontaneous recurrent seizures, are employed to evaluate the antiepileptic potential of novel substances such as levetiracetam and lacosamide (Glien et al., 2002; Behr et al., 2015; Klein et al., 2015; West et al., 2018).

In summary, the use of multiple and complementary seizure models seems to be paramount for detection of novel AEDs. However, combining several *in vivo* models comes with the cost of high number of used animals, and, specifically in case of chronic models, increased animal suffering. This stands in contrast to the 3R principle (reduce, refine, replace) for more ethical use of animals in testing (Russel and Burch, 1959).

*In vitro* models offer the advantage of avoiding animal suffering and of performing several experiments on brain slices from one animal at the same time point. In case of acute seizure models *in vitro*, a plethora of different models has been in use to induce seizure-like events (SLEs), including electrical stimulation (Stasheff et al., 1985, 1989), alteration of the extracellular ion composition (Yaari et al., 1983; Konnerth et al., 1986; Mody et al., 1987; Agopyan and Avoli, 1988; Albrecht et al., 1989; Tancredi et al., 1990), and finally pharmacological intervention (Voskuyl and Albus, 1985; Chesnut and Swann, 1988). Some *in vitro* models were postulated to reflect pharmacoresistance as epileptiform activity in these specific models was not responsive toward standard antiepileptic treatment (Brückner et al., 1999; Albus et al., 2008).

In the present study, we aimed to assess antiepileptic properties across different substance classes using a well-established *in vitro* acute seizure model in rodent brain tissue. A secondary aim was to contribute to the question if this *in vitro* model has the potential to be a valid tool for discovery of AEDs. To that end, we focused on the 4-aminopyridone (4-AP) model of acute seizures. This model has the advantage of stable and spontaneous occurrence of SLEs and a low rate of spreading depolarizations when compared to other models, i.e., the low Mg^2+^ model or electrical stimulation. In previous work, standard AEDs including carbamazepine, topiramate, phenytoin and valproic acid have been investigated in the 4-AP model *in vivo* and *in vitro* (Fueta and Avoli, 1992; Yamaguchi and Rogawski, 1992; Brückner and Heinemann, 2000; D’Antuono et al., 2010). Here, we specifically focused on new-generation AEDs with distinct mechanisms of action. By using a combined electrophysiological and optical approach, we chose to investigate effects of lacosamide (LAC), a sodium channel blocker (Lees et al., 2006; Rogawski et al., 2015), zonisamide (ZNS), for which multiple mechanisms including inhibition of voltage-sensitive T-type calcium currents have been described (Leppik, 2004; Thöne et al., 2008), and finally levetiracetam (LEV), which acts on the synaptic vesicle protein SV2A (Bajjalieh et al., 1994; Lynch et al., 2004).

## Materials and Methods

All experimental procedures were conducted in accordance with the German Animal Welfare Act and the European Directive 2010/63/EU for animal experiments, and with approval of the Institutional Animal Welfare Officer and the responsible local authorities (Landesamt für Gesundheit und Soziales Berlin, T0336/12).

### Combined Hippocampal-Entorhinal Cortex Slice Preparation

All experiments were performed in hippocampal-entorhinal slices from adult, 7–11 week-old, Wistar Han rats (200–350 g). The slices were prepared as described previously (Buchheim et al., 1999) with several modifications. In brief, all animals were decapitated under deep isoflurane anesthesia. The brains were quickly removed and then stored in ice cold artificial cerebrospinal fluid (ACSF) which contained (in mM): NaCl 129.0, KCl 3.0, MgSO_2_ 1.8, CaCl_2_ 1.6, NaH_2_PO_4_ 1.25, NaHCO_3_ 21.0, and glucose 10.0 (pH 7.4), and was continuously oxygenated with carbogen (95% O_2_, 5% CO_2_). Horizontal brain slices (400 μm) were cut using a vibratome (752M Vibroslice, Campden Instruments Ltd., Loughborough, United Kingdom). Slices enclosed the temporal (TC) and perirhinal cortices (PC), the entorhinal cortex (EC), the subiculum (SUB), the dentate gyrus (DG), and the ventral hippocampus. After preparation, slices were placed in a Haas-type interface chamber (Haas et al., 1979) and were continuously perfused (1.5–2.0 ml/min) with prewarmed (35°C) and carbogenated ACSF. In addition, warmed, humified carbogen was directed over the slice surface. Slices were allowed to recover at least 1 h before recordings started on a transparent membrane (0.4 μm Millicell culture plate inserts; Millipore, Bedford, MA, United States).

### Electrophysiological Recordings

Extracellular local field potential (LFP) recordings were carried out with a glass electrode filled with 150 mM NaCl (electrode resistance 1–2 MΩ). Signals were acquired with custom-made amplifiers (10×) connected to an AD converter (Micro 1401 mk II, Cambridge Electronic Design Limited, Cambridge, United Kingdom). Data were recorded and analyzed using Spike2 and Signal (versions 7.00 and 3.07, respectively, Cambridge Electronic Design Limited, Cambridge, United Kingdom) and Matlab (R2014b, MathWorks, Natick, MA, United States) software.

Before assessment of drug effects, slice viability was tested by stimulation of Schaffer collaterals (150 μs, 3–5 mV DC) with a bipolar electrode placed in the stratum radiatum of the distal CA3 area and subsequent recording of population spikes in the CA1 area. Only slices with population spike amplitudes of >2 mV were accepted for further experiments. After viability testing, the stimulation electrode was removed and the recording electrode was placed in the deep layers (IV and V) of the lateral EC.

Seizure like events (SLE) were induced by application of 4-AP (100 μM), a non-selective potassium channel blocker (Rudy, 1988) and a potent proconvulsant compound in the limbic system *in vitro* (Perreault and Avoli, 1991; Yonekawa et al., 1995; Avoli et al., 1996). 4-AP application resulted in occurrence of SLEs that were identified electrographically using the following criteria: (1) decrease in field potential of at least 0.5 mV, (2) duration of the field potential shift of at least 10 s, and (3) superimposition by ripple-like discharges. Slices that did not exhibit SLEs within 45 min of perfusion with 4-AP were excluded.

### Intrinsic Optical Imaging

To monitor spatial propagation of SLEs, we recorded intrinsic optical signals (IOS), which were simultaneously acquired with electrophysiological recordings. Using this methodological approach, the entire slice can be examined non-invasively for onset and propagation of ictal epileptiform activity (Weissinger et al., 2000; Holtkamp et al., 2011). For this purpose, slices were positioned above a curved acrylic glass rod (∅ 8 mm) and then homogeneously illuminated from below by a halogen lamp (KL 1500, Schott, Wiesbaden, Germany). The slices were visualized via an upright binocular light microscope (MS 5, Leica, Bensheim, Germany) and a 2.5× objective (total magnification 12.5×). Video images were acquired by a monocular phototube (Leica, Bensheim, Germany), a CCD camera (8 bit, Sanyo, Osaka, Japan) and an in-house acquisition software. Video signals were converted at a 10 MHz ratio into 320 × 240 pixel images employing a frame-grabber board (pciGrabber-4plus, Phytec, Mainz, Germany). Data were stored on a hard disk of a personal computer for later processing.

All images were processed and analyzed offline using in-house ImageJ/FIJI macros and Matlab software. Images were not saved continuously, but only when triggered by the experimenter in case a SLE became apparent in the electrophysiological recording. Using a circular data buffer, the first image was captured 5–10 s before the electrical onset of the SLE and the recording continued for 50–180 s depending on the duration of the SLE in the electrophysiological recording.

The time course of light transmittance was calculated for each SLE separately as difference (ΔT) in light transmittance between a given image and the control image (mean of the first 20 images in each series, recorded before start of ictal event), and expressed as percentage of the control image intensity (ΔT/T). Results from specific areas (SUB, EC, PC, and TC) were calculated using squared regions of interest (20 × 20 pixels) placed over a given area. During SLEs, ΔT/T typically ranged from 0.4 to 4% whereas noise level fluctuations were below 0.1% within a single region of interest (ROI). For calculation of the SLE area, every pixel within a given anatomical region that reached at least 1% ΔT/T in >9 subsequent images during one SLE was considered to be involved in this SLE. The SLE area was calculated for each region separately and expressed as percentage of the total area for a given anatomical region.

Apart from defined areas of interest, IOS changes over time and space allowed evaluation of SLE onset regions and propagation patterns across the whole slice area. For this purpose, IOS intensity was pseudo color-coded and displayed as videos.

### Assessment of AED Effects

**FIGURE 1:**
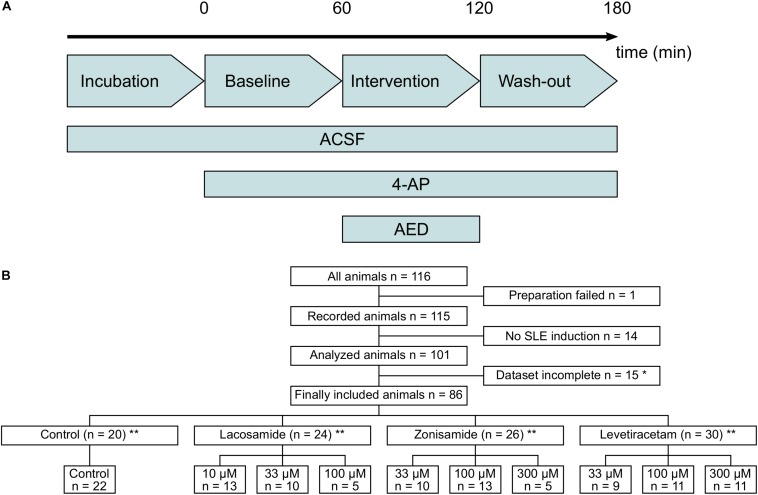
Flow diagram of used laboratory animals and experimental protocol. **(A)** Timeline of the experimental protocol. The duration of baseline, intervention, and wash-out ranged between 40 and 60 min. **(B)** Flow diagram of all laboratory animals used in the study and their allocation to the different intervention groups. Animals with too long preparation, failed SLE induction or incomplete dataset (^*^mostly due to failure of recording before end of the experimental protocol) were excluded from the study. ^∗∗^In few cases, two slices from one animal were used and assigned to different groups. Numbers in lower panel therefore indicate number of slices and in sum might exceed number of animals given above.

Upon establishment of stable SLE amplitude and incidence rate, which occurred after wash-in of 4-AP for ∼40 min, AEDs to be tested were washed in for the duration of 40–60 min. The drug period was followed by a wash-out period of 40–60 min before finishing the experiment. For an overview of the experimental protocol, see Figure 1A. For each phase (baseline, intervention, and wash-out), three to five SLEs were taken for analysis at the end of the respective phase to ensure that possible effects have reached a plateau. Three different concentrations chosen from previous literature were applied for each AED tested: lacosamide: 10, 33, and 100 μM (Lees et al., 2006; May et al., 2015); zonisamide: 33, 100, and 300 μM (Yamaguchi and Rogawski, 1992; Ueda et al., 2003; Thöne et al., 2008); levetiracetam: 33, 100, and 300 μM (Löscher and Hönack, 1993; Margineanu and Klitgaard, 2000; Boido et al., 2010).

### Data Analysis and Presentation

Data were analyzed and figures were processed with R3.5.1 (Vienna, Austria; R Core Team, 2016) and Inkscape 0.92.3 (The Inkscape Project^1^). The responder rate was defined as the ratio of slices with a reduction of SLE frequency of ≥50% during intervention compared to all investigated slices. For comparison between baseline, intervention/second hour and wash-out/third hour, linear mixed-effects models for repeated measurements were fitted followed by ANOVA table calculation (Satterthwaite’s method). *Post hoc* analysis was performed with a Tukey test for multiple comparisons. In case of full SLE block during pharmacological intervention, a two-sided, paired *t*-test was used to compare baseline and wash-out. For comparison of independent groups, one-way ANOVA with *post hoc t*-test (with Benjamini, Hochberg, and Yekutieli adjustment of false discovery rate) was used. In the latter case, *post hoc t*-tests were only calculated between control and intervention groups. To assess the influence of variations in parameters of SLEs at baseline, Pearson’s correlations between the baseline data and the relative effect of the intervention (intervention/baseline) were calculated for SLE frequency and duration. Values of *p* < 0.05 were considered significant. All data are expressed as mean value ± standard deviation.

## Results

From a total of 116 animals used in the study, 86 were included in the final analysis and 30 animals were excluded, mostly due to incomplete recordings and failed SLE induction (Figure 1B). In each experimental group, experiments were performed in tissue from at least nine animals, except of two groups with full SLE block (highest dosages of LAC and ZNS, respectively) in which five animals per group were used. Due to technical reasons and slice storage capacity, in most cases one *in vitro* recording per animal was performed.

### Electrophysiological Recordings

**FIGURE 2:**
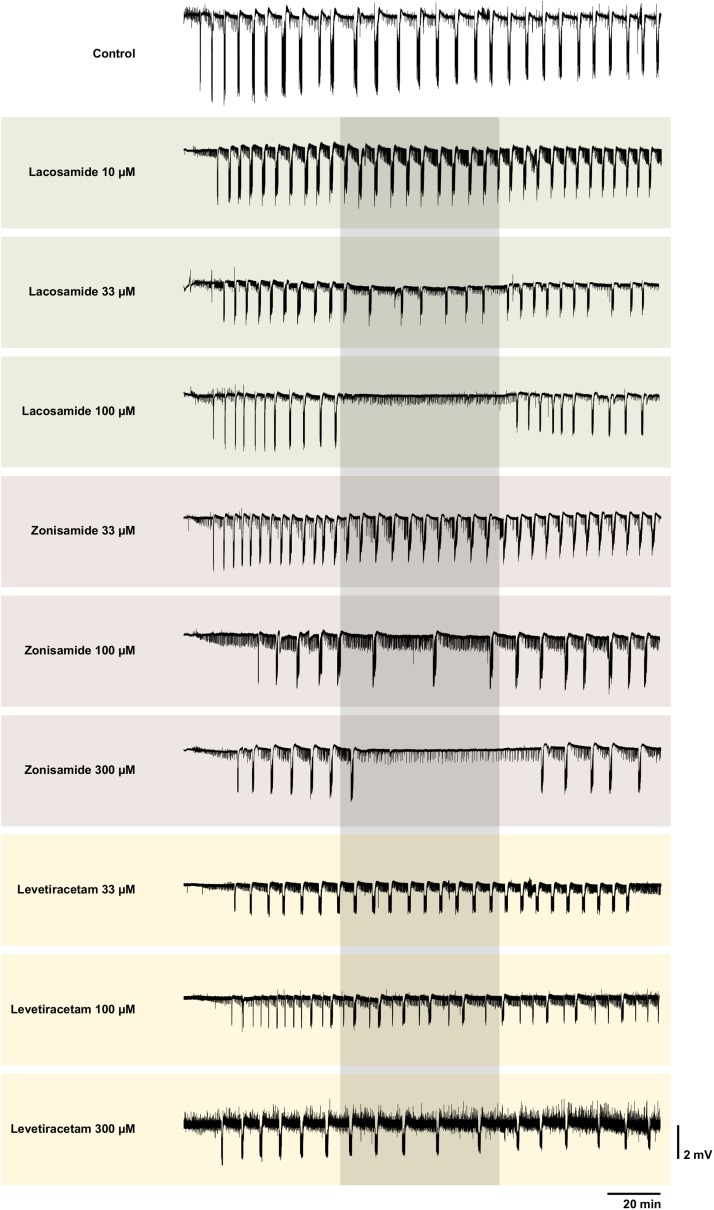
Representative local field potential recordings of control and all intervention groups. Each trace shows a complete recording of the local field potential in EC beginning with the wash-in of 4-AP. The grayish box marks the time window of the intervention with the AED. Note the complete SLE block with LAC 100 and ZNS 300 μM. Though interictal like events were not analyzed systematically, one can clearly see different patterns of their representation. In some recordings, the interictal like events seem to increase during intervention (e.g., LAC 100 μM), in some no visible changes can be observed (e.g., ZNS 100 μM) and in others their frequency seems to be reduced (ZNS 300 μM). Overall there was no obvious clear effect. For better visualization, slow drifts were removed.

**FIGURE 3:**
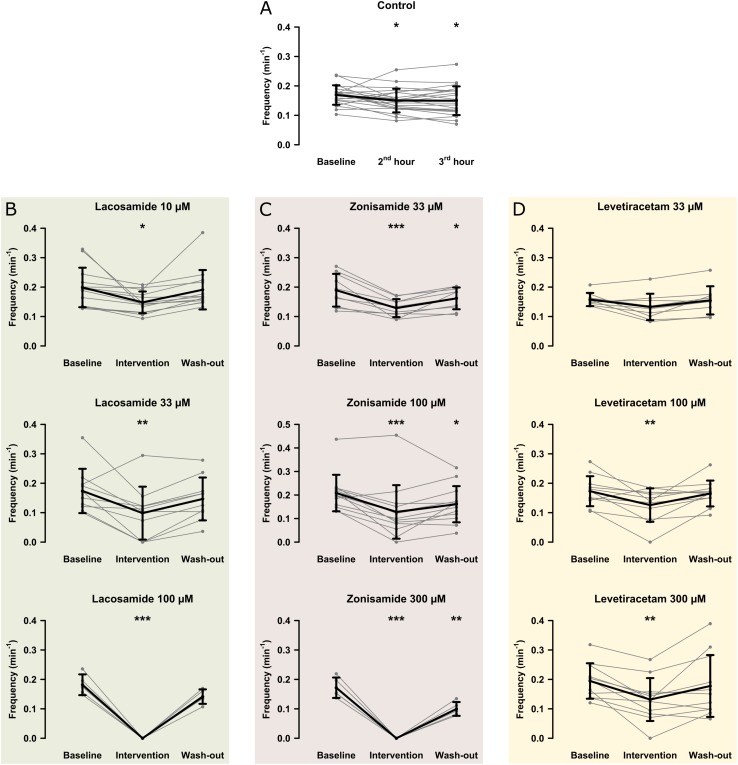
Modulation of the SLE frequency by different AEDs. The scatterplots represent SLE frequency for individual recordings and the superimposed line graphs show the mean ± standard deviation for control **(A)** and the AED **(B–D)** groups. Within the groups, except for LEV 33 μM, SLE frequency significantly decreased from baseline to the end of the intervention (second hour for the control group). Asterisks indicate significant decrease of SLE frequency during and in some cases after intervention compared to baseline (^*^p < 0.05, ^∗∗^*p* < 0.01, ^∗∗∗^*p* < 0.001).

In the first step, we investigated the effects of AEDs on SLE frequency. For an overview of representative recordings in all groups, see Figure 2. In the control group (22 slices, 20 animals), SLEs occurred at a frequency of 0.17 ± 0.03 min^–1^ 1 h after start of 4-AP application, then decreased to 0.15 ± 0.04 min^–1^ in the second hour and remained at this frequency in the last hour of recording (second hour: *p* = 0.041; third hour: *p* = 0.035, Figure 3A). Next, we focused on the effects of LAC, ZNS, and LEV. The effect of the AEDs was independent of SLE frequency and duration at baseline, as there was no significant correlation with the relative changes of these parameters during intervention (tested for LAC 33 μM, ZNS 100 μM, and LEV 100 μM; non-significant correlation coefficients not shown). In case of LAC, all investigated concentrations reduced SLE frequency. In contrast to control experiments in which frequency reduction prevailed in the wash-out phase, the effect of LAC was reversible after wash-out. The effect was also dose-dependent such that SLEs were completely blocked when 100 μM LAC was applied (baseline vs. intervention; 10 μM: 13 slices, *p* = 0.025; 33 μM: 10 slices, *p* = 0.005; 100 μM: 5 slices, *p* < 0.001; Figure 3B). Similar effects were observed upon application of ZNS. Again, reduction of SLE frequency was dose-dependent, and 300 μM ZNS suppressed SLEs completely (baseline vs. intervention; 33 μM: 10 slices, *p* < 0.001; 100 μM: 13 slices, *p* < 0.001; 300 μM: 5 slices, *p* < 0.001; Figure 3C). However, unlike in the LAC group, SLE frequency did not recover completely after wash-out (baseline vs. wash-out; 33 μM: *p* = 0.036; 100 μM: *p* = 0.027; 300 μM: *p* = 0.001).

Compared to LAC and ZNS, the effect of LEV on SLE frequency was markedly smaller. In the lowest concentration (33 μM), no significant effect was observed (baseline vs. intervention: 0.16 ± 0.02 vs. 0.13 ± 0.04 SLE/min, *p* = 0.053, nine slices; Figure 3D). At 100 and 300 μM, LEV application resulted in a reduction of SLE frequency (baseline vs. intervention; 100 μM: 11 slices, *p* = 0.006; 300 μM: 11 slices, *p* = 0.003; Figure 3D), and a complete SLE block occurred only in two slices (one slice in the 100 and 300 μM groups, respectively).

**FIGURE 4:**
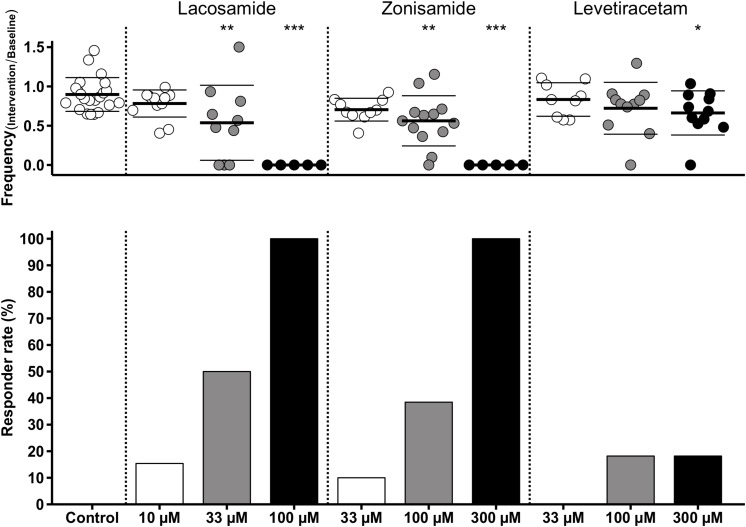
Modulation of SLE frequency by AEDs and responder rates. The upper diagram shows univariate scatterplots of the SLE frequency ratios (intervention/baseline). Compared to the control group, SLE frequency ratio is significantly decreased (indicated by asterisks) in the medium concentration of LAC and ZNS and in the highest concentration of all three AEDs (LAC, ZNS, and LEV). The responder rate (bar graphs in the lower diagram) defined as reduction of SLEs during intervention of at least 50% compared to baseline indicates a stronger anticonvulsant effect of LAC and ZNS vs. LEV.

As stated above, we observed a time-dependent decrease of SLE frequency in control slices. To account for this effect in those experiments with AED application, we performed comparisons of SLE frequency ratios (intervention or second hour of control/baseline) between investigated groups. As expected from intragroup analyses (Figure 3), ratio of SLE frequency was lower after application of LAC (33 and 100 μM) and ZNS (100 and 300 μM) when compared to control (control: 0.90; LAC 33 μM: 0.54, *p* = 0.001; LAC 100 μM: 0, *p* < 0.001; ZNS 100 μM: 0.56, *p* = 0.001; ZNS 300 μM: 0, *p* < 0.001; Figure 4). In the LEV group, only the highest concentration of 300 μM was different from control (LEV 300 μM: 0.66 vs. 0.90, *p* = 0.034). Analysis of the responder rate confirmed that LAC and ZNS displayed a strong and dose-dependent effect on 4-AP-induced SLEs (Figure 4). For a summary of all parameters with regard to SLE frequency, see Supplementary Table S1.

In addition to SLE frequency, we also analyzed SLE duration and the amplitude of the DC shift in all groups except in those with total SLE block (LAC 100 μM, ZNS 300 μM).

In the control group, SLE duration did not change over time (baseline: 53.3 s, after second hour: 56.9 s, after third hour: 52.5 s; *F*(2,42) = 2.98, *p* = 0.061). Overall, drug effects on SLE duration were occasional and not concentration-dependent: ZNS (33 μM) increased SLE duration in a reversible manner (baseline: 60.1 s, intervention: 68.1 s, *p* = 0.040; wash-out: 60.6 s, *p* = 0.986), but the effect was not different from control (*p* = 0.544) when comparing effect ratios (see above). Application of LEV (300 μM) resulted in a continuous decrease of SLE duration (baseline: 58.2 s, intervention: 46.2 s, *p* = 0.031; wash-out: 37.7 s, *p* < 0.001), which was the only effect different from control (*p* = 0.015) when comparing effect ratios. The remaining groups showed no alterations of SLE duration. The amplitude of the DC shift decreased over time in the control group (baseline: 1.64 mV, second hour: 1.52 mV, *p* = 0.025; third hour: 1.43 mV, *p* < 0.001) but also when LAC 33 μM, ZNS 33 μM, or LEV 300 μM were applied. These effects were not different from controls (*F*(7,85) = 1.81, *p* = 0.096). For a summary of all parameters of SLE duration and amplitude of the DC shift, see also Supplementary Table S1.

### Intrinsic Optical Signals

**FIGURE 5:**
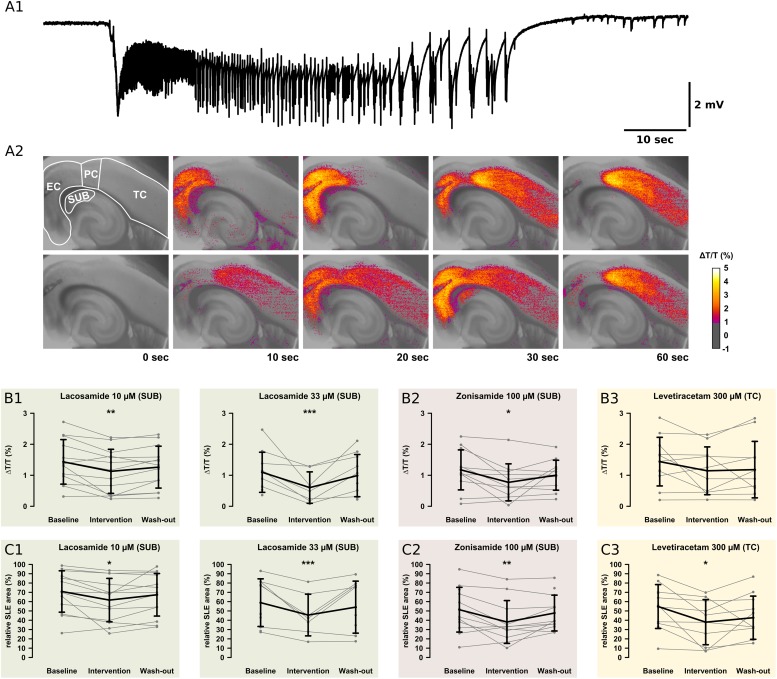
Recordings of the IOS. For analysis of the onset zone, spread, and local intensity of the SLEs, the IOS was recorded simultaneously to local field potentials. As described in previous studies, the SLEs started in different regions within the same slice. Note the relatively small area of the SUB. **(A1)** The electrophysiological recording shows a representative SLE from the EC. After the onset with a sharp transient there is a tonic phase resting about 10 s. This goes over into the clonic phase, which lasts about 50 s. **(A2)** The pictures show the color-coded IOS intensity over time during an SLE. In the upper row, the SLE starts in the EC and then continuously spreads mainly to the PC and TC. In the lower row, the SLE starts in the PC and then spreads vice versa to the TC and EC. In the SUB, LAC **(B1,C1)** and ZNS **(B2,C2)** reduce the IOS intensity and the size of the area the SLE is spreading to. The third AED, LEV, reduces the SLE area but not IOS intensity in the TC **(B3,C3)**. All effects are reversed at the end of the wash-out. Asterisks indicate significant decrease of IOS intensity or SLE area compared to baseline (^*^p < 0.05, ^∗∗^*p* < 0.01, ^∗∗∗^*p* < 0.001).

Intrinsic optical signals was acquired in order to investigate drug effects on SLE onset, intensity, and propagation. All experiments except those with SLE block upon AED application (LAC 100 μM, ZNS 300 μM) were included. Analysis was performed separately in four predefined subregions (SUB, EC, PC, and TC, see Figure 5A). In the majority of slices, SLE onset was located in the EC and TC areas as described before (Weissinger et al., 2000, 2017; Figure 5A).

In the control group, IOS intensity (ΔT/T) remained unchanged in all investigated regions (Supplementary Table S2). Also after AED application, ΔT/T did not change in most regions except the SUB. Here, LAC (10 μM, 33 μM) and ZNS (100 μM), but not LEV, markedly reduced ΔT/T (LAC 10 μM: baseline: 1.43%, intervention: 1.13%, *p* = 0.001; LAC 33 μM: baseline: 1.10%, intervention: 0.38%, *p* < 0.001; ZNS: baseline: 1.17%, intervention: 0.78%, *p* = 0.016; Figures 5B,C). Intergroup analysis revealed differences in the intervention-to-baseline ratio of ΔT/T in the SUB between drug and control groups (*F*(7,84) = 2.82; *p* = 0.011), however, significance level was not reached in *post hoc* tests.

The area affected by the SLE was calculated for each region separately and expressed as percentage of the investigated region. In the control group, no differences in the SLE area between successive time points were detectable. Similar to ΔT/T, application of LAC (10 μM, 33 μM) or ZNS (100 μM) resulted in a reduction of the SLE area exclusively in the SUB (LAC 10 μM: baseline: 70.9%, intervention: 61.7%, *p* = 0.016; LAC 33 μM: baseline: 58.9%, intervention: 37.8%, *p* < 0.001; ZNS 100 μM: baseline: 51.3%, intervention: 40.2%, *p* = 0.008; Figures 5C1,C2). In contrast, application of LEV did not affect the SUB but reduced SLE area in the TC when applied at a concentration of 300 μM (baseline: 54.8%, intervention 37.6%, *p* = 0.025; Figure 5C3). Intergroup analyses detected differences between drug and control groups in the SUB (*p* = 0.005), which, however, could not be confirmed in *post hoc* tests (Supplementary Table S3). For a summary of all investigated IOS parameters, see Supplementary Tables S2, S3.

## Discussion

In the present study, we assessed antiepileptic effects of selected new-generation AEDs with distinct mechanisms of action in the 4-AP model of acute seizures *in vitro*. We demonstrated that in addition to standard AEDs tested previously, the 4-AP model *in vitro* was also able to detect antiepileptic effects in new-generation AEDs (LAC, ZNS, LEV) at concentrations approximate to those observed in patients (Schulz et al., 2012).

Simultaneous electrophysiological and optical recordings in acute hippocampal slices revealed SLE frequency as the most sensitive parameter. While all applied AEDs induced a robust decrease of SLE frequency, the effect on SLE duration and amplitude of the DC shift was small and not compelling and the IOS effects were region- and compound-selective. In contrast to our data, Lees et al. (2006) reported a reduced duration and a diminished DC shift of SLEs induced by 4-AP in the visual cortex after wash-in of LAC (2006), but only at a high dose (100 μM) that completely blocked SLEs in our experiments. The results suggest that AED sensitivity might vary across distinct cortical regions. With regard to LEV, our observations are in line with previous *in vivo* studies demonstrating reduced SLE frequency but not altered SLE duration (Glien et al., 2002; Lévesque et al., 2015).

Beside SLEs, we observed interictal like events in most of the recordings, which are thought to represent interictal epileptiform activity *in vivo*. As the clinical relevance and the diagnostic value of interictal activity are discussed controversially (Usui et al., 2008; Jacobs et al., 2010), in this *in vitro* study, we renounced to systematically analyze interictal like events and focused on the thorough analysis of SLEs.

Of note, the investigated new-generation AEDs primarily did not modulate other SLE properties than frequency and thus rather seem to affect their induction but not maintenance of ictal activity. Importantly, gap junctions are involved in the control of seizure duration *in vivo* (Gajda et al., 2003), and none of the applied AEDs has been described to modulate gap junctions.

While both LAC and ZNS displayed a robust and dose-dependent antiepileptic effect and were capable of completely blocking SLEs, the effect of LEV in this model was smaller. Still, in contrast to lacking effects in the PTZ/MES models *in vivo*, the 4-AP model *in vitro* was able to detect an antiepileptic effect of LEV within the therapeutic concentration range albeit it was rather small. A direct comparison of *in vivo* and *in vitro* drug concentrations is always challenging, mainly because of different mechanisms of drug uptake (blood-brain-barrier, permeability of tissue, active transport). The AED concentrations chosen here ranging from the lower limit to a maximum of 1.3–1.8 times above the upper limit of the therapeutic range in humans (Schulz et al., 2012). This is in accordance with a previous study that suggested that the effective concentration *in vivo* and *in vitro* might be in the same range (Heinemann et al., 1985).

In contrast to LAC and LEV, ZNS displayed an antiepileptic effect that was persistent after wash-out of the substance. This observation suggests that ZNS might induce long-lasting changes in the intrinsic excitability that outlive the direct substance effect. A possible explanation could be a long-lasting reduction in intracellular pH induced by carbonic anhydrase inhibition (but see Thöne et al., 2008).

As mentioned above, IOS effects were confined to specific regions, i.e., LAC and ZNS reduced IOS intensity and the area affected by the SLEs specifically in the SUB. IOS intensity was calculated as mean per ROI, therefore reduction of the affected area alone could have led to reduced calculated IOS intensity by a higher pixel number of a given ROI not reaching detection threshold. In conclusion, the observed reduced IOS intensity might represent an effect of technical limitations (ROI size, video resolution) rather than a real reduction. Irrespective of that, the specific effect in the SUB is intriguing. Previous studies underpinned the crucial role of the SUB in the induction of SLEs in the 4-AP model (Benini and Avoli, 2005; Herrington et al., 2015), which might explain the high sensitivity of this area to LAC and ZNS.

*In vitro* seizure models in brain slices offer the advantage of performing several experiments in one animal and simultaneously avoiding the animal burden produced by *in vivo* seizure events. In our study, we performed 1–2 *in vitro* experiments per animal, which seems rather low and was mostly due to limited storage and recording capacities in our facility. However, in case of larger technical capacities as often provided in drug screening units, up to 10 experiments per animal seem to be a realistic estimate, which is determined mostly by biological constraints, i.e., brain size of the investigated species.

Together with previous literature, our findings suggest that the 4-AP model *in vitro* is sensitive for the detection of antiepileptic properties across substances with various mechanisms of action. This applies to standard AEDs (Fueta and Avoli, 1992; D’Antuono et al., 2010), new-generation AEDs (this work) as well as future potential antiepileptic substances. Dóczi et al. (1999) demonstrated that AMPA receptor antagonists can dose-dependently modulate the frequency and duration of interictal discharges in the neocortex of adult rats in the 4-AP model *in vitro*. However, there is currently no study, which verifies these findings with the clinically used AMPA receptor antagonist perampanel. Enhancement of GABAergic inhibition by benzodiazepine derivatives was effective against epileptiform activity (evoked epileptiform burst activity, SLEs) in two independent studies *in vitro* (Marinelli et al., 2000; Ridler et al., 2018), which is in line with the involvement of GABAergic interneurons in the synchronous activity during SLEs in the 4-AP model (Grosser et al., 2014). Finally, in cultured hippocampal neurons, KCNQ potassium channel openers suppressed epileptiform activity induced by 4-AP (Kobayashi et al., 2008). In the current study we did not involve a negative control using a known proconvulsant substance. For a possible screening tool of the anticonvulsant properties, the detection of a negative effect of the screened substances would be essential. Adding the GABA-A receptor antagonist bicuculline to the 4-AP model has been shown to make SLEs resistant against standard anticonvulsant drugs like phenytoin, carbamazepine, valproic acid, or phenobarbital (Brückner et al., 1999). Future studies should test if this effect is consistent also with new-generation anticonvulsant drugs.

As mentioned above, there are a number of relevant *in vitro* models of acute seizures. Compared to these models, the 4-AP model applied here offers the advantage of stable conditions regarding SLE morphology and frequency over several hours, low rate of spreading depolarizations, and ictal activity which clearly can be distinguished from interictal discharges. In contrast, the low Mg^2+^ model has a high occurrence rate of spreading depolarizations, and the SLEs are not stable for more than an hour (Mody et al., 1987). Furthermore, the SLEs are often resistant to standard AEDs such as carbamazepine and valproic acid. This might be advantageous if one is looking for novel treatment options of pharmacoresistance, but is a hindrance for exploratory screening (Dreier et al., 1998; Dost and Rundfeldt, 2000; Arias and Bowlby, 2005). The high K^+^ model also has a high rate of spreading depolarizations, and in rats, the epileptiform discharges are very variable with respect to morphology and frequency (Traynelis and Dingledine, 1988). This disadvantage can be avoided by using another species, such as mice (Lopantsev et al., 2009). Thereby, depending on the aim of this study and the applied substances, a proper choice of the chosen species might be necessary. The GABA-A receptor antagonists gabazine and picrotoxine convert hippocampal sharp wave-ripple-complexes into recurrent epileptiform discharges (Behrens et al., 2007). There is little data on the effects of AEDs or long-term stability of the SLEs. Taken together, the 4-AP model seems to be advantageous in terms of SLE induction and stability, however, further studies are necessary for comparison of its potential with other *in vitro* models.

A major limitation of acute seizure models *in vivo* and *in vitro* is the lacking representation of epileptic conditions occurring after epileptogenesis in neuronal networks. Therefore, these models are not capable of representing features of the drug-resistant brain. This might in part explain the failed translation of findings from these animal models to patients with epilepsy and in particular with drug-resistant epilepsy. In a systematic screening approach designed to overcome this enigma, a combination of both acute and chronic epilepsy models and the use of translationally more relevant *ex vivo* human tissue (e.g., from epilepsy surgery) is suggested. In this respect, the 4-AP model *in vitro* might present as a possible candidate model with high sensitivity to detect antiepileptic properties, which could be included in an exploratory screening line. In this first step, this would reduce the number of *in vivo* experiments. However, following preselection, *in vivo* approaches might be unavoidable in subsequent testing in chronic models of epilepsy and pharmacoresistance.

## Data Availability

The datasets generated for this study are available on request to the corresponding author.

## Ethics Statement

All experimental procedures were conducted in accordance with the German Animal Welfare Act and the European Directive 2010/63/EU for animal experiments, and with approval of the Institutional Animal Welfare Officer and the responsible local authorities (Landesamt für Gesundheit und Soziales Berlin, T0336/12).

## Author Contributions

MH, HH, PF, and RD contributed to the conception and design of the study. HH and RD performed the electrophysiological and optical recordings, and analyzed the data. MW analyzed the data and performed the statistical analysis. MW wrote the first draft of the manuscript. PF revised the Introduction and Discussion sections of the manuscript. All authors read and approved the final version of the manuscript for submission.

## Conflict of Interest Statement

The authors declare that the research was conducted in the absence of any commercial or financial relationships that could be construed as a potential conflict of interest.

## Footnotes

1 https://inkscape.org
