# Supplementary material for: The 4-Aminopyridine Model of Acute Seizures in vitro Elucidates Efficacy of New Antiepileptic Drugs
Source: Front Neurosci. 2019 Jun 27;13:677. doi: 10.3389/fnins.2019.00677 (PMC6610309; doi:10.3389/fnins.2019.00677)
Supplement: Supplementary file 1 [file Table_1.DOCX]

Table S1. Summary of all analyzed SLE parameters: frequency, duration, and amplitude of the DC shift

| Group | Slices | SLE Frequency (min^-1^) | | | Intra group | Inter group^A^ |
| --- | --- | --- | --- | --- | --- | --- |
|  |  | Baseline | Intervention | Wash out |  | (vs. control) |
| Control | 22 | 0.169 ± 0.033 | 0.150 ± 0.040 | 0.150 ± 0.049 | F(2,42) = 4.34; p = 0.019  **base – inter: p = 0.041**  **base – wash: p = 0.035**  inter – wash: p = 0.998 | ANOVA:  F(9,99) = 10.01  p < 0.001 |
| LAC 10 µM | 13 | 0.199 ± 0.067 | 0.148 ± 0.037 | 0.191 ± 0.067 | F(2,24) = 4.58; p = 0.021  **base – inter: p = 0.025**  base – wash: p = 0.904  inter – wash: p = 0.064 | p = 0.248 |
| LAC 33 µM | 10 | 0.174 ± 0.075 | 0.098 ± 0.090 | 0.147 ± 0.073 | F(2,18) = 6.80; p = 0.006  **base – inter: p = 0.005**  base – wash: p = 0.407  inter – wash: p = 0.077 | **p = 0.001** |
| LAC 100 µM | 5 | 0.182 ± 0.035 | 0 | 0.142 ± 0.025 | F(2,8) = 88.31; p < 0.001  **base – inter: p < 0.001**  base – wash: p = 0.054  **inter – wash: p < 0.001** | **p < 0.001** |
| ZNS 33 µM | 10 | 0.189 ± 0.056 | 0.129 ± 0.031 | 0.162 ± 0.037 | F(2,18) = 17.84; p < 0.001  **base – inter: p < 0.001**  **base – wash: p = 0.036**  **inter – wash: p = 0.012** | p = 0.090 |
| ZNS 100 µM | 13 | 0.208 ± 0.078 | 0.128 ± 0.114 | 0.161 ± 0.077 | F(2,24) = 11.23; p < 0.001  **base – inter: p < 0.001**  **base – wash: p = 0.027**  inter – wash: p = 0.154 | **p = 0.001** |
| ZNS 300 µM | 5 | 0.172 ± 0.035 | 0 | 0.100 ± 0.023 | F(2,8) = 97.31; p < 0.001  **base – inter: p < 0.001**  **base – wash: p = 0.001**  **inter – wash: p < 0.001** | **p < 0.001** |
| LEV 33 µM | 9 | 0.158 ± 0.022 | 0.133 ± 0.045 | 0.155 ± 0.048 | F(2,16) = 3.91; p = 0.041  base – inter: p = 0.053  base – wash: p = 0.957  inter – wash: p = 0.090 | p = 0.546 |
| LEV 100 µM | 11 | 0.173 ± 0.051 | 0.126 ± 0.057 | 0.165 ± 0.044 | F(2,20) = 7.17; p = 0.005  **base – inter: p = 0.006**  base – wash: p = 0.831  **inter – wash: p = 0.021** | p = 0.100 |
| LEV 300 µM | 11 | 0.195 ± 0.060 | 0.132 ± 0.073 | 0.178 ± 0.105 | F(2,20) = 7.80; p = 0.003  **base – inter: p = 0.003**  base – wash: p = 0.570  **inter – wash: p = 0.029** | **p = 0.034** |
|  |  |  |  |  |  |  |
| Group | Slices | SLE Duration (seconds) | | | Intra group | Inter group^A^ |
|  |  | Baseline | Intervention | Wash out |  | (vs. control) |
| Control | 22 | 53.33 ± 14.64 | 56.89 ± 14.31 | 52.45 ± 10.94 | F(2,42) = 2.98; p = 0.061 | ANOVA:  F(7,85) = 3.09  p = 0.006 |
| LAC 10 µM | 13 | 54.73 ± 15.46 | 58.52 ± 14.62 | 52.11 ± 11.56 | F(2,24) = 1.96; p = 0.162 | p = 0.965 |
| LAC 33 µM | 10 | 52.53 ± 15.27 | 41.70 ± 15.99 | 49.47 ± 6.53 | F(2,15.9) = 2.27; p = 0.135 | **p = 0.030** |
| LAC 100 µM | 5 | 51.53 ± 28.81 | - | 54.47 ± 19.41 | two-sided, paired t-test  baseline vs. wash out  p = 0.568 | - |
| ZNS 33 µM | 10 | 60.07 ± 16.53 | 68.07 ± 17.30 | 60.56 ± 16.70 | F(2,18) = 4.47; p = 0.027  **base – inter: p = 0.040**  base – wash: p = 0.986  inter – wash: p = 0.055 | p = 0.544 |
| ZNS 100 µM | 13 | 53.35 ± 14.07 | 53.03 ± 15.74 | 48.82 ± 15.01 | F(2,23.1) = 1.97, p = 0.163 | p = 0.392 |
| ZNS 300 µM | 5 | 50.97 ± 11.39 | - | 48.90 ± 13.21 | two-sided, paired t-test  baseline vs. wash out  p = 0.373 | - |
| LEV 33 µM | 9 | 53.21 ± 12.27 | 51.77 ± 14.34 | 48.48 ± 11.48 | F(2,16) = 3.42; p = 0.058 | p = 0.255 |
| LEV 100 µM | 11 | 51.72 ± 17.22 | 48.94 ± 14.38 | 46.43 ± 10.60 | F(2,19.1) = 3.32; p = 0.058 | p = 0.255 |
| LEV 300 µM | 11 | 58.23 ± 20.62 | 46.24 ± 15.45 | 37.70 ± 17.78 | F(2,19.2) = 11.05; p < 0.001  **base – inter: p = 0.031**  **base – wash: p < 0.001**  inter – wash: p = 0.220 | **p = 0.015** |
|  |  |  |  |  |  |  |
| Group | Slices | Amplitude of DC-shift (mV) | | | Intra group | Inter group^A^ |
|  |  | Baseline | Intervention | Wash out |  | (vs. control) |
| Control | 22 | 1.642 ± 0.891 | 1.516 ± 0.859 | 1.425 ± 0.816 | F(2,42) = 11.15; p < 0.001  **base – inter: p = 0.025**  **base – wash: p < 0.001**  inter – wash: p = 0.131 | ANOVA:  F(7,85) = 1.81  p = 0.096 |
| LAC 10 µM | 13 | 2.696 ± 0.925 | 2.591 ± 1.005 | 2.459 ± 1.134 | F(2,24) = 2.46; p = 0.107 |  |
| LAC 33 µM | 10 | 1.973 ± 0.819 | 2.223 ± 0.896 | 1.552 ± 0.938 | F(2,15.02) = 13.72; p < 0.001  base – inter: p = 0.479  **base – wash: p < 0.001**  **inter – wash: p = 0.014** |  |
| LAC 100 µM | 5 | 2.745 ± 0.902 | - | 2.293 ± 1.028 | two-sided, paired t-test  baseline vs. wash out  p = 0.063 | - |
| ZNS 33 µM | 10 | 2.154 ± 0.607 | 1.816 ± 0.787 | 1.632 ± 0.715 | F(2,18) = 18.74; p < 0.001  **base – inter: p = 0.003**  **base – wash: p < 0.001**  inter – wash: p = 0.113 |  |
| ZNS 100 µM | 13 | 2.031 ± 0.499 | 1.912 ± 0.624 | 1.897 ± 0.594 | F(2,23.04) = 1.03, p = 0.373 |  |
| ZNS 300 µM | 5 | 1.975 ± 0.507 | - | 1.604 ± 0.703 | two-sided, paired t-test  baseline vs. wash out  p = 0.102 | - |
| LEV 33 µM | 9 | 0.833 ± 0.395 | 0.763 ± 0.400 | 0.795 ± 0.447 | F(2,16) = 1.34, p = 0.289 |  |
| LEV 100 µM | 11 | 1.129 ± 0.574 | 1.024 ± 0.418 | 1.023 ± 0.391 | F(2,19.12) = 0.95, p = 0.403 |  |
| LEV 300 µM | 11 | 1.222 ± 0.694 | 1.086 ± 0.705 | 0.964 ± 0.630 | F(2,19.01) = 16, p < 0.001  **base – inter: p = 0.004**  **base – wash: p < 0.001**  inter – wash: p = 0.280 |  |

^A^: Inter-group comparison of ratios (intervention/baseline) of frequency and duration of SLEs as well as amplitude of the DC shift between all groups, post-hoc tests were performed between control and AED groups.
