# Supplementary material for: The 4-Aminopyridine Model of Acute Seizures in vitro Elucidates Efficacy of New Antiepileptic Drugs
Source: Front Neurosci. 2019 Jun 27;13:677. doi: 10.3389/fnins.2019.00677 (PMC6610309; doi:10.3389/fnins.2019.00677)
Supplement: Supplementary file 2 [file Table_2.DOCX]

Table S2. IOS intensity (ΔT/T) in the investigated regions for all study groups

| **SUB** | | | | | | |
| --- | --- | --- | --- | --- | --- | --- |
| Group | Slices | IOS intensity ΔT/T (%) | | | Intra group | Inter group^A^ |
|  |  | Baseline | Intervention | Wash out |  | (vs. control) |
| Control | 22 | 1.056 ± 0.464 | 0.943 ± 0.383 | 0.927 ± 0.373 | F(2,42) = 2.69, p = 0.079 | ANOVA:  F(7,84) = 2.82  p = 0.011 |
| LAC 10 µM | 13 | 1.432 ± 0.718 | 1.127 ± 0.710 | 1.262 ± 0.675 | F(2,24) = 8.62, p = 0.002  **base – inter: p = 0.001**  base – wash: p = 0.073  inter – wash: p = 0.181 | p = 0.538 |
| LAC 33 µM | 10 | 1.096 ± 0.647 | 0.603 ± 0.507 | 0.989 ± 0.681 | F(2,15.08) = 13.08, p < 0.001  **base – inter: p < 0.001**  base – wash: p = 0.685  **inter – wash: p = 0.002** | p = 0.063 |
| LAC 100 µM | 5 | 1.106 ± 0.462 | - | 1.067 ± 0.499 | two-sided, paired t-test  baseline vs. wash out  p = 0.584 | - |
| ZNS 33 µM | 10 | 0.890 ± 0.511 | 0.863 ± 0.612 | 0.891 ± 0.505 | F(2,18) = 0.04, p = 0.962 | p = 0.279 |
| ZNS 100 µM | 12 | 1.173 ± 0.645 | 0.771 ± 0.597 | 1.002 ± 0.483 | F(2,21.19) = 4.68, p = 0.021  **base – inter: p = 0.016**  base – wash: p = 0.371  inter – wash: p = 0.220 | p = 0.665 |
| ZNS 300 µM | 5 | 0.902 ± 0.449 | - | 0.698 ± 0.490 | two-sided, paired t-test  baseline vs. wash out  p = 0.119 | - |
| LEV 33 µM | 9 | 0.985 ± 0.344 | 1.166 ± 0.393 | 1.057 ± 0.243 | F(2,16) = 2.36, p = 0.127 | p = 0.279 |
| LEV 100 µM | 11 | 0.989 ± 0.488 | 0.865 ± 0.209 | 0.928 ± 0.373 | F(2,18.29) = 0.21, p = 0.809 | p = 0.665 |
| LEV 300 µM | 11 | 0.595 ± 0.297 | 0.553 ± 0.342 | 0.484 ± 0.295 | F(2,19.11) = 1.42, p = 0.267 | p = 0.811 |
|  |  |  |  |  |  |  |
| **EC** | | | | | | |
| Group | Slices | IOS intensity ΔT/T (%) | | | Intra group | Inter group^A^ |
|  |  | Baseline | Intervention | Wash out |  | (vs. control) |
| Control | 22 | 3.241 ± 1.029 | 3.258 ± 0.991 | 3.123 ± 1.073 | F(2,42) = 1.17, p = 0.321 | ANOVA:  F(7,84) = 1.38  p = 0.225 |
| LAC 10 µM | 13 | 3.500 ± 0.948 | 3.641 ± 1.253 | 3.678 ± 1.056 | F(2,24) = 0.57, p = 0.575 |  |
| LAC 33 µM | 10 | 3.183 ± 1.102 | 3.124 ± 0.904 | 3.289 ± 1.069 | F(2,15.03) = 4.90, p = 0.023  base – inter: p = 0.062  base – wash: p = 0.803  **inter – wash: p = 0.021** |  |
| LAC 100 µM | 5 | 2.950 ± 0.976 | - | 3.246 ± 1.221 | two-sided, paired t-test  baseline vs. wash out  p = 0.130 |  |
| ZNS 33 µM | 10 | 3.034 ± 0.674 | 3.34 ± 0.901 | 3.338 ± 1.100 | F(2,18) = 2.28, p = 0.131 |  |
| ZNS 100 µM | 12 | 3.321 ± 0.972 | 3.14 ± 0.903 | 3.183 ± 0.886 | F(2,21.07) = 0.49, p = 0.622 |  |
| ZNS 300 µM | 5 | 2.870 ± 0.603 | - | 3.234 ± 1.280 | two-sided, paired t-test  baseline vs. wash out  p = 0.376 |  |
| LEV 33 µM | 9 | 2.926 ± 0.708 | 2.906 ± 0.954 | 2.871 ± 0.704 | F(2,16) = 0.06, p = 0.943 |  |
| LEV 100 µM | 11 | 2.455 ± 0.616 | 2.524 ± 0.445 | 2.559 ± 0.667 | F(2,19.02) = 0.28, p = 0.759 |  |
| LEV 300 µM | 11 | 2.44 ± 0.820 | 2.495 ± 0.837 | 2.431 ± 0.800 | F(2,19.09) = 0.20, p = 0.819 |  |
|  |  |  |  |  |  |  |
| **PC** | | | | | | |
| Group | Slices | IOS intensity ΔT/T (%) | | | Intra group | Inter group^A^ |
|  |  | Baseline | Intervention | Wash out |  | (vs. control) |
| Control | 22 | 2.056 ± 1.223 | 2.211 ± 1.469 | 2.235 ± 1.503 | F(2,42) = 1.47, p = 0.242 | ANOVA:  F(7,84) = 1.33  p = 0.245 |
| LAC 10 µM | 13 | 1.850 ± 0.924 | 1.890 ± 1.055 | 2.097 ± 1.035 | F(2,24) = 1.03, p = 0.372 |  |
| LAC 33 µM | 10 | 1.976 ± 0.904 | 1.687 ± 0.910 | 2.510 ± 1.101 | F(2,15.64) = 4.06, p = 0.038  base – inter: p = 0.512  base – wash: p = 0.186  **inter – wash: p = 0.036** |  |
| LAC 100 µM | 5 | 1.554 ± 1.331 | - | 1.271 ± 1.252 | two-sided, paired t-test  baseline vs. wash out  p = 0.573 |  |
| ZNS 33 µM | 10 | 2.021 ± 1.036 | 2.192 ± 1.072 | 2.371 ± 1.184 | F(2,18) = 1.24, p = 0.313 |  |
| ZNS 100 µM | 12 | 2.636 ± 1.347 | 2.565 ± 1.493 | 2.368 ± 1.444 | F(2,21.04) = 0.88, p = 0.428 |  |
| ZNS 300 µM | 5 | 2.148 ± 0.256 | - | 2.566 ± 0.434 | two-sided, paired t-test  baseline vs. wash out  p = 0.102 |  |
| LEV 33 µM | 9 | 1.586 ± 0.747 | 1.894 ± 1.066 | 1.551 ± 0.759 | F(2,16) = 2.61, p = 0.104 |  |
| LEV 100 µM | 11 | 1.160 ± 0.531 | 1.403 ± 0.829 | 1.498 ± 0.656 | F(2,19.21) = 2.46, p = 0.112 |  |
| LEV 300 µM | 11 | 1.326 ± 0.725 | 1.119 ± 0.755 | 1.028 ± 0.732 | F(2,19.14) = 1.69, p = 0.211 |  |
|  |  |  |  |  |  |  |
| **TC** | | | | | | |
| Group | Slices | IOS intensity ΔT/T (%) | | | Intra group | Inter group^A^ |
|  |  | Baseline | Intervention | Wash out |  | (vs. control) |
| Control | 22 | 2.046 ± 1.210 | 2.253 ± 1.520 | 2.234 ± 1.520 | F(2,42) = 1.82, p = 0.174 | ANOVA:  F(7,84) = 1.12  p = 0.358 |
| LAC 10 µM | 13 | 1.495 ± 0.845 | 1.591 ± 1.127 | 1.704 ± 1.052 | F(2,24) = 0.74, p = 0.486 |  |
| LAC 33 µM | 10 | 1.992 ± 1.162 | 1.289 ± 1.157 | 2.618 ± 1.557 | F(2,15.26) = 5.38, p = 0.017  base – inter: p = 0.235  base – wash: p = 0.213  **inter – wash: p = 0.013** |  |
| LAC 100 µM | 5 | 1.345 ± 1.460 | - | 0.960 ± 0.802 | two-sided, paired t-test  baseline vs. wash out  p = 0.377 |  |
| ZNS 33 µM | 10 | 2.122 ± 1.624 | 2.067 ± 1.364 | 2.194 ± 1.401 | F(2,18) = 0.11, p = 0.893 |  |
| ZNS 100 µM | 12 | 2.324 ± 1.344 | 2.211 ± 1.404 | 2.099 ± 1.469 | F(2,21.05) = 0.81, p = 0.456 |  |
| ZNS 300 µM | 5 | 2.252 ± 1.151 | - | 2.198 ± 0.897 | two-sided, paired t-test  baseline vs. wash out  p = 0.863 |  |
| LEV 33 µM | 9 | 1.684 ± 0.549 | 1.883 ± 0.745 | 1.877 ± 0.579 | F(2,16) = 1.70, p = 0.214 |  |
| LEV 100 µM | 11 | 1.702 ± 1.059 | 1.618 ± 1.060 | 1.790 ± 1.321 | F(2,19.09) = 0.27, p = 0.765 |  |
| LEV 300 µM | 11 | 1.440 ± 0.784 | 1.144 ± 0.771 | 1.182 ± 0.910 | F(2,19.13) = 1.94, p = 0.171 |  |

^A^: Inter-group comparison of ratios (intervention/baseline) of frequency and duration of SLEs as well as amplitude of the DC shift between all groups, post-hoc tests were performed between control and AED groups.
