# Supplementary material for: The 4-Aminopyridine Model of Acute Seizures in vitro Elucidates Efficacy of New Antiepileptic Drugs
Source: Front Neurosci. 2019 Jun 27;13:677. doi: 10.3389/fnins.2019.00677 (PMC6610309; doi:10.3389/fnins.2019.00677)
Supplement: Supplementary file 3 [file Table_3.DOCX]

Table S3. Relative SLE area as calculated with the IOS in the investigated regions for all study groups

| **SUB** | | | | | | |
| --- | --- | --- | --- | --- | --- | --- |
| Group | Slices | Relative SLE area (%) | | | Intra group | Inter group^A^ |
|  |  | Baseline | Intervention | Wash out |  | (vs. control) |
| Control | 22 | 54.50 ± 21.40 | 51.84 ± 18.89 | 50.84 ± 19.81 | F(2,42) = 1.17, p = 0.321 | ANOVA:  F(7,84) = 3.22  p = 0.005 |
| LAC 10 µM | 13 | 70.91 ± 22.23 | 61.67 ± 23.32 | 67.37 ± 22.86 | F(2,24) = 4.66, p = 0.020  **base – inter: p = 0.016**  base – wash: p = 0.488  inter – wash: p = 0.171 | p = 0.293 |
| LAC 33 µM | 10 | 58.90 ± 25.61 | 45.59 ± 22.39 | 54.08 ± 27.97 | F(2,15.1) = 10.97, p = 0.001  **base – inter: p < 0.001**  base – wash: p = 0.470  **inter – wash: p = 0.008** | p = 0.089 |
| LAC 100 µM | 5 | 69.16 ± 16.08 | - | 61.28 ± 13.63 | two-sided, paired t-test  baseline vs. wash out  p = 0.090 | **-** |
| ZNS 33 µM | 10 | 63.21 ± 18.88 | 59.80 ± 25.29 | 60.50 ± 23.12 | F(2,18) = 0.55, p = 0.589 | p = 0.378 |
| ZNS 100 µM | 12 | 51.32 ± 24.03 | 38.13 ± 23.02 | 47.75 ± 19.26 | F(2,21.02) = 5.91, p = 0.009  **base – inter: p = 0.008**  base – wash: p = 0.512  inter – wash: p = 0.078 | p = 0.232 |
| ZNS 300 µM | 5 | 37.68 ± 8.74 | - | 33.37 ± 13.77 | two-sided, paired t-test  baseline vs. wash out  p = 0.296 | **-** |
| LEV 33 µM | 9 | 44.88 ± 13.11 | 51.73 ± 17.35 | 55.02 ± 14.20 | F(2,16) = 3.55, p = 0.053 | p = 0.293 |
| LEV 100 µM | 11 | 42.74 ± 13.65 | 37.32 ± 14.90 | 40.88 ± 15.24 | F(2,19.06) = 0.78, p = 0.473 | p = 0.378 |
| LEV 300 µM | 11 | 26.91 ± 14.77 | 30.57 ± 10.70 | 26.98 ± 13.17 | F(2,19.02) = 0.42, p = 0.663 | p = 0.216 |
|  |  |  |  |  |  |  |
| **EC** | | | | | | |
| Group | Slices | Relative SLE area (%) | | | Intra group | Inter group^A^ |
|  |  | Baseline | Intervention | Wash out |  | (vs. control) |
| Control | 22 | 79.82 ± 13.90 | 79.33 ± 12.67 | 77.55 ± 13.59 | F(2,42) = 1.58, p = 0.218 | ANOVA:  F(7,84) = 0.59  p = 0.765 |
| LAC 10 µM | 13 | 77.22 ± 10.83 | 76.77 ± 9.41 | 76.46 ± 9.93 | F(2,24) = 0.15, p = 0.861 |  |
| LAC 33 µM | 10 | 77.31 ± 7.68 | 76.73 ± 4.15 | 75.08 ± 7.86 | F(2,14.91) = 3.33, p = 0.064 |  |
| LAC 100 µM | 5 | 84.58 ± 3.05 | - | 80.28 ± 7.34 | two-sided, paired t-test  baseline vs. wash out  p = 0.269 |  |
| ZNS 33 µM | 10 | 81.25 ± 9.57 | 79.10 ± 16.80 | 77.54 ± 15.51 | F(2,18) = 0.67, p = 0.522 |  |
| ZNS 100 µM | 12 | 77.10 ± 11.22 | 74.50 ± 9.11 | 75.97 ± 7.93 | F(2,21.05) = 0.48, p = 0.624 |  |
| ZNS 300 µM | 5 | 78.66 ± 5.30 | - | 78.27 ± 10.00 | two-sided, paired t-test  baseline vs. wash out  p = 0.930 |  |
| LEV 33 µM | 9 | 77.61 ± 10.04 | 75.86 ± 12.40 | 77.67 ± 6.28 | F(2,16) = 0.20, p = 0.825 |  |
| LEV 100 µM | 11 | 77.91 ± 6.63 | 79.41 ± 5.69 | 77.08 ± 7.45 | F(2,19.31) = 0.71, p = 0.504 |  |
| LEV 300 µM | 11 | 69.96 ± 20.20 | 69.85 ± 18.07 | 72.36 ± 19.4 | F(2,19.13) = 0.15, p = 0.864 |  |
|  |  |  |  |  |  |  |
| **PC** | | | | | | |
| Group | Slices | Relative SLE area (%) | | | Intra group | Inter group^A^ |
|  |  | Baseline | Intervention | Wash out |  | (vs. control) |
| Control | 22 | 67.77 ± 21.17 | 69.34 ± 20.84 | 69.94 ± 20.72 | F(2,42) = 0.50, p = 0.609 | ANOVA:  F(7,84) = 0.90  p = 0.514 |
| LAC 10 µM | 13 | 61.45 ± 20.96 | 63.15 ± 19.67 | 61.12 ± 18.14 | F(2,24) = 0.14, p = 0.868 |  |
| LAC 33 µM | 10 | 64.45 ± 17.60 | 54.61 ± 19.57 | 67.95 ± 13.97 | F(2,15.55) = 2.91, p = 0.084 |  |
| LAC 100 µM | 5 | 60.23 ± 30.76 | - | 52.80 ± 36.52 | two-sided, paired t-test  baseline vs. wash out  p = 0.438 |  |
| ZNS 33 µM | 10 | 69.16 ± 23.87 | 69.76 ± 26.21 | 69.04 ± 25.73 | F(2,18) = 0.05, p = 0.950 |  |
| ZNS 100 µM | 12 | 74.81 ± 19.97 | 69.58 ± 18.97 | 70.92 ± 16.58 | F(2,21.09) = 1.39, p = 0.271 |  |
| ZNS 300 µM | 5 | 82.04 ± 5.29 | - | 87.22 ± 6.50 | two-sided, paired t-test  baseline vs. wash out  **p = 0.043** |  |
| LEV 33 µM | 9 | 68.53 ± 17.78 | 66.83 ± 21.20 | 63.15 ± 18.56 | F(2,16) = 0.45, p = 0.647 |  |
| LEV 100 µM | 11 | 56.65 ± 20.31 | 58.76 ± 25.74 | 61.85 ± 14.59 | F(2,19.13) = 0.92, p = 0.417 |  |
| LEV 300 µM | 11 | 60.30 ± 29.70 | 51.04 ± 27.76 | 54.64 ± 27.47 | F(2,19.25) = 0.88, p = 0.433 |  |
|  |  |  |  |  |  |  |
| **TC** | | | | | | |
| Group | Slices | Relative SLE area (%) | | | Intra group | Inter group^A^ |
|  |  | Baseline | Intervention | Wash out |  | (vs. control) |
| Control | 22 | 55.38 ± 17.49 | 56.30 ± 16.48 | 58.05 ± 16.59 | F(2,42) = 0.83, p = 0.442 | ANOVA:  F(7,84) = 1.61  p = 0.143 |
| LAC 10 µM | 13 | 41.65 ± 22.81 | 43.92 ± 21.97 | 46.78 ± 21.12 | F(2,24) = 0.74, p = 0.488 |  |
| LAC 33 µM | 10 | 59.14 ± 26.90 | 41.82 ± 29.55 | 65.13 ± 23.84 | F(2,15.33) = 3.93, p = 0.042  base – inter: p = 0.163  base – wash: p = 0.601  **inter – wash: p = 0.034** |  |
| LAC 100 µM | 5 | 44.24 ± 29.16 | - | 34.70 ± 28.75 | two-sided, paired t-test  baseline vs. wash out  p = 0.362 |  |
| ZNS 33 µM | 10 | 44.63 ± 26.42 | 47.09 ± 27.56 | 47.90 ± 26.52 | F(2,18) = 1.36, p = 0.282 |  |
| ZNS 100 µM | 12 | 59.00 ± 30.33 | 53.60 ± 31.21 | 54.02 ± 27.74 | F(2,21.05) = 1.79, p = 0.192 |  |
| ZNS 300 µM | 5 | 62.15 ± 28.39 | - | 54.06 ± 23.53 | two-sided, paired t-test  baseline vs. wash out  p = 0.257 |  |
| LEV 33 µM | 9 | 55.59 ± 9.53 | 59.57 ± 15.10 | 60.83 ± 12.61 | F(2,16) = 0.71, p = 0.506 |  |
| LEV 100 µM | 11 | 59.80 ± 24.67 | 53.45 ± 29.60 | 58.82 ± 23.76 | F(2,19.05) = 1.36, p = 0.281 |  |
| LEV 300 µM | 11 | 54.74 ± 23.60 | 37.94 ± 24.27 | 42.73 ± 23.23 | F(2,19.23) = 4.45, p = 0.026  **base – inter: p = 0.025**  base – wash: p = 0.120  inter – wash: p = 0.670 |  |

^A^: Inter-group comparison of ratios (intervention/baseline) of frequency and duration of SLEs as well as amplitude of the DC shift between all groups, post-hoc tests were performed between control and AED groups.
